# Facile Synthesis of Ce-MOF for the Removal of Phosphate, Fluoride, and Arsenic

**DOI:** 10.3390/nano13233048

**Published:** 2023-11-29

**Authors:** Lili Zhang, Decheng Mao, Yining Qu, Xiaohong Chen, Jindi Zhang, Mengyang Huang, Jiaqiang Wang

**Affiliations:** 1 School of Chemistry and Resources Engineering, Honghe University, Mengzi 661100, China; zhanglili@uoh.edu.cn (L.Z.); zhangjindi@uoh.edu.cn (J.Z.); 2 School of Materials and Energy, Yunnan University, Kunming 650091, China; mao296468691@outlook.com; 3 School of Chemical Sciences & Technology, Yunnan University, Kunming 650091, China; yiningqu@foxmail.com; 4 Institute of International Rivers and Eco-Security, Yunnan University, Kunming 650091, China; xiaohongchen18@foxmail.com

**Keywords:** phosphate, fluoride, arsenic (V), adsorption, Ce-MOF, wastewater

## Abstract

Ce-MOF was synthesized by a solvothermal synthesis method and was used to simultaneously remove phosphate, fluoride and arsenic (V) from water by adsorption. Ce-MOF was characterized by a nitrogen adsorption–desorption isotherm, scanning electron microscopy, and infrared spectroscopy. The effects of initial concentration, adsorption time, adsorption temperature, pH value and adsorbent on the adsorption properties were investigated. A Langmuir isotherm model was used to fit the adsorption data, and the adsorption capacity of phosphate, fluoride, and arsenic (V) was calculated to be 41.2 mg·g^−1^, 101.8 mg·g^−1^ and 33.3 mg·g^−1^, respectively. Compared with the existing commercially available CeO_2_ and other MOFs, Ce-MOF has a much higher adsorption capacity. Furthermore, after two reuses, the performance of the adsorbent was almost unchanged, indicating it is a stable adsorbent and has good application potential in the field of wastewater treatment.

## 1. Introduction

The problem of water pollution involves various sources of pollution, including industrial wastewater, agricultural pollution, urban sewage, and so on. The harmful substances released by these pollution sources, such as heavy metals, organic matter, and nutrients, not only damage the water ecosystem but also have a serious impact on human health. Therefore, it is imperative to deeply study and solve the world water pollution problem [1]. 

The occurrence of phosphorus, fluorine, and arsenic in natural water has caused severe environmental pollution. High levels of phosphorus can lead to the eutrophication of water bodies. In addition, phosphorus also poses significant harm to organisms. Phosphorus in the environment can inhibit the action of cholinesterase in vertebrates, such as humans and livestock, affect nervous system function, and cause poisoning or even death. For marine organisms, organic phosphorus can activate latent pathogens in shrimp bodies, leading to the death of fish and shrimp in the ocean [2,3]. Arsenic has been listed as the first type of carcinogen by the World Health Organization. Arsenides are prone to accumulate in the human body, and long-term, low-dose intake can lead to chronic arsenic poisoning, leading to neurological diseases such as neurasthenia and neuritis. The WHO currently recommends an arsenic limit of 10 μg L^−1^ for drinking water. However, it is estimated that more than 100 million people have arsenic levels in drinking water that exceed the 10 μg L^−1^ level issued by the WHO [4]. Excessive intake of drinking water with excessive arsenic content can lead to acute arsenic poisoning, damaging the gastrointestinal system, respiratory system, skin, and nervous system. In severe cases, it can lead to neurological abnormalities, breathing difficulties, heart failure, and death. In addition, prolonged exposure of human skin to arsenic-contaminated water can lead to damage to skin keratin, and in severe cases, it can lead to skin cancer [5,6]. Fluorine causes fluorosis when it is excessive, and there is no specific drug for the treatment of fluorosis. Excessive fluoride content in drinking water can lead to developmental disorders in children’s teeth, leading to dental fluorosis. In addition, if the fluoride content in drinking water is too high, in addition to causing dental fluorosis, it can also deposit on bones, leading to abnormal bone development, causing symptoms such as overall bone and joint pain, fatigue, and osteoporosis and bone sclerosis. After an external impact, fractures are prone to occur, and in severe cases, deformation or even paralysis of the entire body’s bones and joints can occur, resulting in loss of labor ability [7,8].

Therefore, how to restore polluted wastewater is an urgent problem for us.

Many methods have been developed to remove phosphate, fluoride, and arsenic from aqueous systems, including coagulation/filtration, ion exchange, membrane technologies, chemical precipitation, and adsorption [9,10]. At present, there are four commonly used methods for phosphorus removal: the phosphate precipitation method, biological method, ion exchange method, and redox method. According to different water quality and treatment requirements, targeted treatment methods need to be selected to avoid unnecessary water pollution in the scientific phosphorus removal process. Arsenic removal generally includes coagulation, adsorption, ion exchange, and other methods. The commonly used fluoride removal processes currently include traditional activated alumina fluoride removal, reverse osmosis membrane treatment, and molecular sieve fluoride removal [11]. 

Sludge disposal problems exist in coagulation. Membrane technology and the high cost of the ion exchange method, as well as the disadvantage of its frequent need for maintenance, keeps it from being widely applied in water pollution control. Chemical precipitation may have drawbacks such as the excessive use of toxic substances, which is not conducive to cleaner production. Compared with other technologies, adsorption is widely used because of its simple, sustainable, economically viable, and highly efficient nature [12]. As we know, metal ions, including iron, zirconium, and manganese, and especially rare earth elements in the form of oxides, show an excellent removal performance towards hazardous anions [13,14,15,16,17,18]. Among these metal oxides, cerium oxide is one of the most abundant and least expensive rare earth metal oxides [19,20,21]. Cerium oxide particles are coarse, with a particle diameter of 100–500 nm, and highly crystalline. As we all know, CeO_2_ is one of the most reactive rare earth metal oxides, with a superior oxygen storage capacity, and can be used as a good accelerator or carrier in industrial catalytic processes. Cerium oxide also has good electrical conductivity and dispersion at ambient temperature [22,23]. In recent years, cerium-based sorbents have been paid more attention for water purification due to their excellent adsorption performance in the removal of anionic contaminants (e.g., phosphate, fluoride, and arsenic). It was found that Ce-based materials, in particular, Ce (III) species, play a major role in binding with phosphate [24]. 

Metal–organic skeletons (MOF) are multifunctional nanoporous material consisting of a crystal network structure of metal ions or metal clusters coordinated with organic ligands [25]. In this framework, metals and organic molecules are coordinated, which creates a structural framework with both metal activity, organic flexibility, the selectivity of functional groups, and other physicochemical properties. Due to the pore structure and large specific surface area, enough active sites are created for ion adsorption; as a result, MOFs have a great advantage in adsorption elimination [26,27]. MOFs have been widely used in separation, catalysis, adsorption, sensors, luminescence, and magnetic fields [28,29,30,31]. 

As a material for pollutant removal in water treatment, MOFs have many advantages. Their specific surface area is large enough to reflect their excellent adsorption capacity. They have many active sites, and pollutants can be chemically adsorbed. MOFs can be prepared on a large scale. Some are water stable. An MOF, protonated MIL-101, which has a high adsorption capacity (194 mg g^−1^), was described for the removal of dyes [32]. In addition to their adjustable pore structures, MOFs also have a rich functional group composition, which makes them suitable for the selective adsorption of different metal ions. For example, some MOF materials containing nickel oxide or iron ions can efficiently adsorb heavy metal ions, such as lead, chromium, and nickel, in water and have better adsorption properties in a wide pH range. [33,34]

Recently, research has shown that different MOF compounds have shown their superiority in the field of adsorption. For example, a new type of Zn-MOF was synthesized by using a low-cost, low-toxicity zinc acetate dihydrate metal salt as the metal source and cheap H2pda and 5-amino-1H-tetrazole as organic ligands. The maximum phosphate adsorption capacity of Zn-MOF-500 prepared by carbonization is 226.07 mg g^−1^. However, the carbonization process increases the process of product preparation, and the uncarbonized Zn-MOF is not suitable for large-scale production because of its poor thermal stability [35].

In the field of fluorine removal, an Al-MOF-5 metal-organic framework material was synthesized by a hydrothermal method. The maximum adsorption capacity of the material was 46.08 mg g^−1^ at 39.85 °C. The adsorption mechanism of Al-MOF-5 is the ion exchange between OH^−^ and F^−^. At the same time, Al-MOF-5 can achieve better adsorption effects in different pH environments and can be recycled many times. However, the durability and adsorption capacity of Al-MOF-5 cannot meet current operation requirements; additionally, its synthesis cost is high, and it cannot be used in actual production and life [36].

In addition, a new type of Fe-Co organic skeleton adsorption nanostructure (MOF-74) has been synthesized by a solvothermal method. The diameter of the MOF-74 nanoparticles is between 60 and 80 nm, and their specific surface area is 147.82 m^2^ g^−1^. When the molar ratio of Fe:Co is 2:1, the adsorption capacity of arsenic is the best, and the maximum adsorption capacity of As (III) and As(V) is 266.52 and 292.29 mg g^−1^, respectively. However, when the solution contains a higher concentration of phosphate, it significantly affects the adsorption effect of MOF-74 on arsenic [37].

MIL-100 (Fe) was successfully used for the removal of As (V), with an adsorption capacity of 110 mg g^−1^ [38]. At the same time, metal–organic scaffolds (MOFs) have also attracted attention in the field of defluorination. Their special surface area, various functional sites, and adjustable pore structure make them capable of detecting fluorine ions while having the potential to remove fluorine ions from water. In terms of phosphate removal, there have also been studies to develop many MOF adsorbents [39]. Due to the unique nature of Ce, interest in Ce-MOFs has increased significantly in the research community. Compared with other metal frames, the synthesis time of Ce-MOFs is short, and their energy consumption is relatively low. Therefore, it is feasible to use Ce-MOFs as adsorbents for polluted water. It has been shown that Ce-MOFs are used to remove phosphate from eutrophication water, showing an adsorption capacity of 179 mg g^−1^, and are superior to other porous adsorbents. In addition, due to the electropositive character of Ce^4+^, it has a strong binding affinity for F^−^, so Ce-MOFs can effectively remove F^−^. Although the adsorption properties of Ce-MOF composites have been studied extensively, there are still some limitations [40,41].

Ce-MOFs, as a kind of derivative, have made remarkable achievements in different fields. The present study shows that derivatives based on Ce-MOFs can be obtained by different preparation methods. In the field of removing Congo red pollutants from water, a kind of adsorbent Ce-MOF was synthesized by a microwave method. The Ce-MOF material was used in the preparation of high-performance electrode materials. In addition to the traditional dispersing method using a binder and ethanol solvent, a method of making a Ce-MOF electrode by ultrasound has also been developed. Otherwise, at present, most Ce-MOF derivatives are still synthesized by more traditional and mature hydrothermal methods [42].

Although the synthesis method of Ce-MOF is still relatively simple, its application field is very wide. In addition to the applications of adsorbents and electrode materials mentioned above, Ce-MOF has applications in many fields, such as photocatalytic materials, antioxidant materials, crystal luminescent materials, catalysts, and radiation protection [43]. 

Ce-MOF-5 synthesized by different methods shows significant differences in morphology and fluorine adsorption performance. The effect of the initial concentration, pH value, and adsorption time on the adsorption effect of F^−^ was also determined. Ce-MOF-66 and Ce-MOF-808 were prepared by a facile solvothermal method with the modulation of organic ligands, and they have shown good selectivity for As (III) and As (V) in an aqueous solution [38].

An ideal adsorbent should possess several advantages such as rapid adsorption kinetics, high adsorption capacity, excellent stability, and a strong affinity towards phosphate, fluoride, and arsenic. However, at present, studies have rarely been reported that have been conducted on the removal of these three ions by Ce-MOF. Inspired by the above considerations, we aimed to synthesize rectangular parallelepiped Ce-MOF with the solvothermal process and applied it to remove phosphate, fluoride, and arsenic (V) from aqueous systems. In this study, Ce-MOF was synthesized by a solvothermal synthesis method. It was characterized and used for the adsorption of phosphate, fluoride, and arsenic (V) in water at the same time. This work provides a new concept for the removal of these three ions simultaneously.

## 2. Experiment

### 2.1. Synthesis of Cerium Terephthalic Acid Complex

The materials and the experimental conditions that were used for each process during the synthesis of the adsorbent and adsorption process are showed in the Supplementary Information (Text S1).

The synthesis conditions of the complexes were optimized by changing the amount of solution (V_DMF_ = 40 mL, 60 mL, 80 mL, 100 mL, Table S1), reaction temperature (T = 100 °C, 150 °C, 180 °C, 200 °C, Table S2), and reaction time (t = 1 d, 2 d, 3 d, 4 d, Table S3). The synthesized materials were used for the removal of simulated phosphate-containing wastewater (1 mg L^−1^) to identify the appropriate preparation method of complexes, where 2.6 g Ce(NO_3_)_3_·6H_2_O, 4.34 g H_3_BTC and 60 mL DMF were added in Teflon bottle. The mixture solution was stirred for a while, then treated at 150 °C for 72 h. After reaction, the mixture was naturally cooled, then centrifuged and washed several times with DMF and ethanol until the product changed to light yellow. Subsequently, the mixture was purified with ethanol at 90 °C for one day, then filtered, washed, and dried to obtain the complex.

### 2.2. Adsorption Experiments

Phosphate and arsenic removal from different samples was performed through batch experiments. Solid samples weighing between 0.1 and 0.5 g were added into a big beaker with 2 L of solution containing 1 mg L^−1^ phosphate and arsenic. The pH of the solution was adjusted by 1 mg L^−1^ hydrochloric acid (HCl) and sodium hydroxide (NaOH) and measured with a pH meter (Lei Ci, Shanghai, China) without buffer before adsorption. Sodium dihydrogen phosphate (NaH_2_PO_4_) and H_5_As_3_O_10_ were used as the phosphorus and arsenic sources, respectively. Fifteen milliliters of deionized water and various amounts of Ce-MOF were mixed in the vortex mixer for about 5 min and then injected into 2 L of phosphate and arsenic solution. The sub-sample of the solution was collected into the syringe and filtered with a 0.25 μm filter disk into a sample tube properly. Then, the phosphate and arsenic concentrations of the filtered solutions were analyzed according to molybdenum antimony spectrophotometry. The concentration of fluoride in the aqueous solutions before and after adsorption was determined by the ion-selective potential method. The treated fluorine-containing solution was filtered with a 0.25 μm microporous filter disk into a capacity bottle in volumes of 5 mL to 100 mL. The pH was adjusted by HCl and NaOH. Trisodium citrate–sodium nitrate buffer solution was used as an ion buffer solution. Then, the treated solution was poured into a polytetrafluoroethylene beaker. The equilibrium potential value was read after the system stabilized under the action of the electrode. The fluorine content in the solution was found according to the standard curve. The fluorine standard solution removal rate and fluoride removal capacity were also calculated.

The removal efficiency and adsorption capacity (mg L^−1^) in a batch experiment were calculated by the following equations:

(1) $$\mathrm{Removal}\;\mathrm{efficiency}=\frac{\left(C_{0}-C_{e}\right)}{C_{0}}\times 100\%$$

(2) $$\mathrm{Adsorption}\;\mathrm{capacity}=\frac{V(C_{0}-C_{e})}{m}$$

where *C*_0_ (mmol L^−1^) and *C_e_* (mmol L^−1^) are the initial concentration and the concentration of these three ions at equilibrium, respectively. *V* (mL) and *m* (g) denote the volume of the aqueous solution and the weight of Ce-MOF adsorbent, respectively.

### 2.3. Characterizations and Measurements

Pore size distributions, BET surface areas, and pore volumes were measured by nitrogen adsorption–desorption using a NOVA 2000e gas sorption analyzer (Quantachrome Corp, Boynton Beach, FL, USA). The total pore volume and pore-size distribution were evaluated by the Barett–Joyner–Halenda method from the adsorption branch of the isotherm and using a cylindrical model. Prior to the analysis, the samples were degassed at 150 °C for 1 h. SEM images were taken on a FEIQuanta200FEG (FEI Company, Hillsboro, OR, USA) microscope at an accelerating voltage of 15 kV with the pressure in the sample chamber set at 1 Torr. FT-IR measurements were performed on a Thermo Nicolet AVATAR FT-IR 360(Thermo Fisher Scientific, Waltham, MA, USA) instrument. Potassium bromide pellets containing 0.5% of the catalysts were used in FTIR experiments, and 125 scans were accumulated for each spectrum in transmission, at a spectral resolution of 4 cm^−1^. The spectrum of dry KBr was taken for background subtraction.

## 3. Results and Discussion 

### 3.1. Powder X-ray Diffraction (XRD) of Ce-MOF

**Figure 1:**
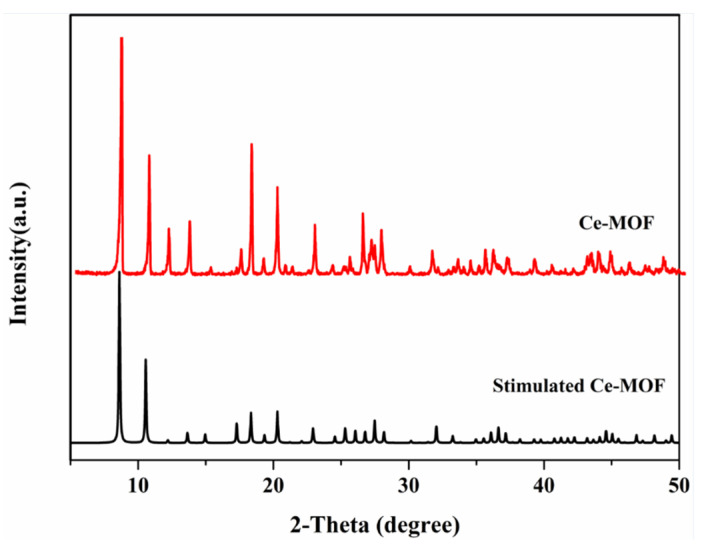
XRD patterns of Ce-MOF.

Powder X-ray diffraction (XRD) experiments were conducted on a D/max-3B spectrometer, and scans were made in the 2θ range 10–50° with a scan rate of 10°/min (wide-angle diffraction). XRD patterns (Figure 1) showed that Ce-MOF was successfully synthesized. XRD patterns (Figure S1) show that the synthesized material was not cerium oxide.

### 3.2. Nitrogen Adsorption–Desorption Isotherms of Ce-MOF

**Figure 2:**
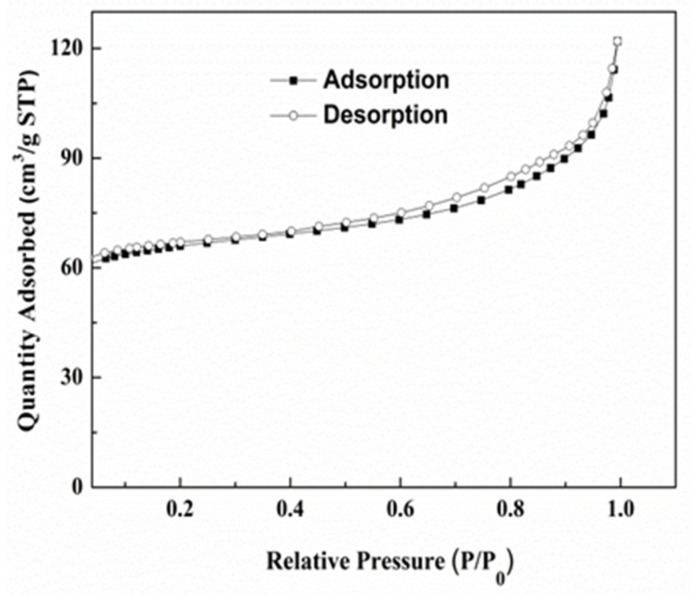
Nitrogen adsorption–desorption isotherms of cerium dioxide (Ce-MOF).

The nitrogen adsorption–desorption isotherm (Figure 2) of Ce-MOF exhibited a typical isotherm of a type IV curve, indicating the presence of mesoporous materials. There is a hysteresis loop in the high-pressure zone (nitrogen relative pressure *p/p*_0_ = 0.6–1.0) due to the incompletely reversible adsorption–desorption caused by mesoporous capillary condensation. At lower relative pressures, the curve is approximately linear, indicating a single-layer adsorption on the mesoporous surface. The BET surface area and pore size of the complex are 222.4 m^2^ g^−1^ and 10.7 nm, respectively. The nitrogen adsorption–desorption isotherm of Ce-MOF proves that it can play a full role in the experiment. At the same time, the synthesized complex has the characteristics of recycling, and its success reflects the greenness of the experiment.

### 3.3. SEM Analysis

**Figure 3:**
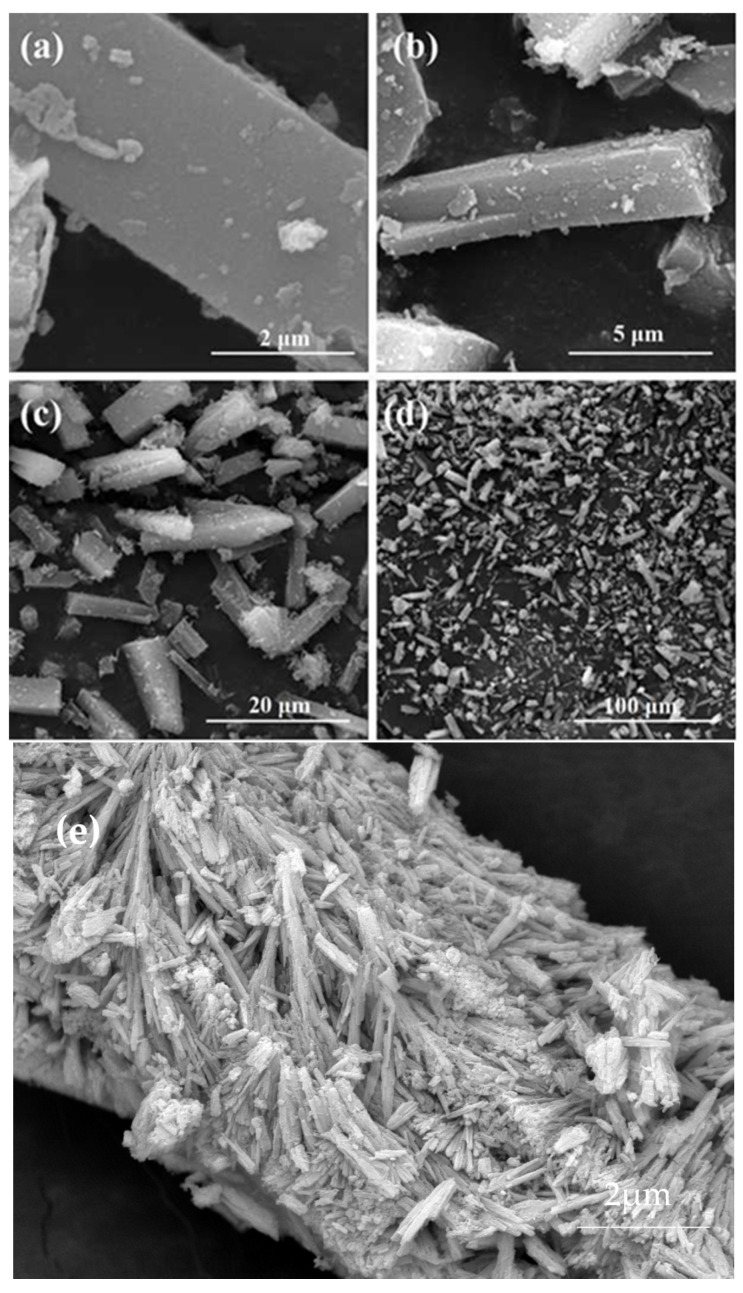
(**a**–**d**) Scanning electron microscopy (SEM) of Ce-MOF at different scales. (**e**) Scanning electron microscopy (SEM) after adsorption.

It can be seen from the scanning electron microscopy in Figure 3 that the morphology of the complex is uniform with a rectangular parallelepiped, with a width of 3–5 μm and a length of 15–20 μm. After adsorption, the SEM scan showed that many rod-like particles were attached. After adsorption, the SEM images of Ce-MOF showed that there were many rod-like particles attached to the originally smooth material. Nevertheless, it can still be seen that the overall structure is consistent with that of the Ce-MOF material before adsorption.

### 3.4. FTIR Spectroscopy Analysis

**Figure 4:**
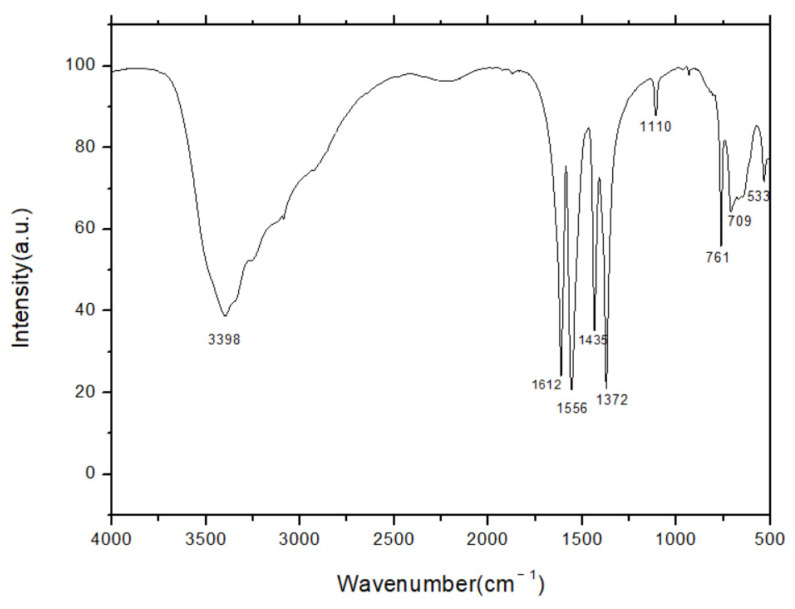
Fourier transform infrared spectroscopy (FTIR) of Ce-MOF.

FTIR was employed to obtain further structural information about the Ce-MOF. Figure 4 presents FT-IR spectra of the Ce-MOF between 500 and 4000 cm^−1^. It can be seen that the material has an obvious vibrational absorption at 1600 cm^−1^ to 1400 cm^−1^, indicating the presence of a benzene ring. There is a significant vibration absorption at about 1700 cm^−1^, which shows the presence of a carboxylate group and can be assigned to the C=O stretch. The above analysis shows that the structure of benzene tricarboxylic acid was present in the cerium complexes.

### 3.5. Effect of Operating Parameters on Phosphate Removal

#### 3.5.1. Effect of Initial Anion Concentration

**Figure 5:**
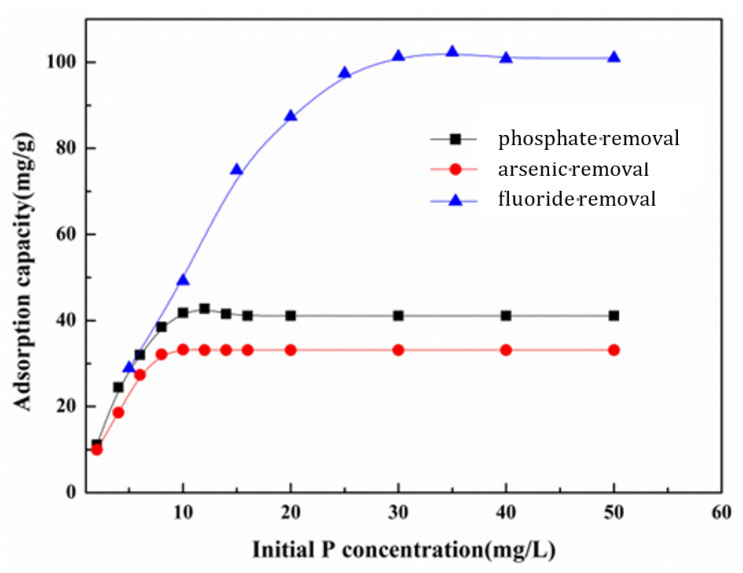
Effect of initial phosphate concentration on phosphate, fluoride, and arsenic removal.

To explore the effect of the initial phosphate, fluoride, and arsenic concentration on Ce-MOF, batch tests were conducted. Under the conditions of a pH of 7 and a reaction temperature of 25 degrees, the initial concentration of anions increased from 2 mg L^−1^ to 50 mg L^−1^, and the additional amounts of Ce-MOF were 0.2 g L^−1^. Figure 5 shows the adsorption capacity as a function of the initial concentration. It can be seen that with an increase in the initial concentration, the adsorption capacity gradually increases, and then gradually tends to balance. In the beginning, the adsorbent had a low adsorption capacity because the number of adsorption sites on the materials was much higher than that of the phosphate, fluoride, and arsenic ions in solution. When the initial concentration increased, the amount of anions also increased, and the high concentration of adsorbate produced a high driving force promoting the migration of anions to the adsorbent surface, leading to an increase in the adsorption capacity. However, when the initial concentration increased to a certain amount, the adsorption amount was slightly increased due to the almost-filled active sites. The phosphate adsorption over adsorbents was substantially saturated at this moment. It can be seen that when the initial concentrations of phosphate, fluoride, and arsenic solution were about 12 mg L^−1^, 35 mg L^−1^, and 10 mg L^−1^, respectively, the adsorption capacity of the adsorbent reached equilibrium at values of 42.8 mg g^−1^, 102.3 mg g^−1^ and 33.2 mg g^−1^, respectively. 

#### 3.5.2. Effect of pH

**Figure 6:**
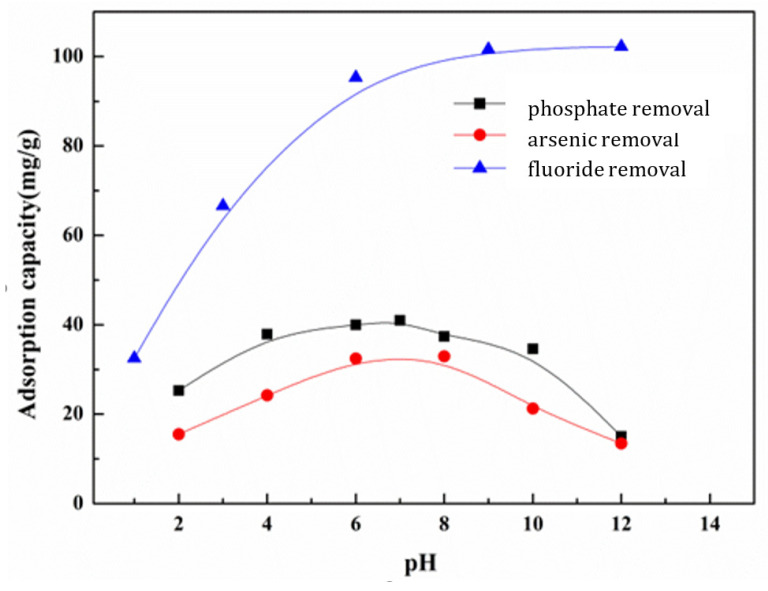
Effect of pH on phosphate, fluoride, and arsenic removal.

To explore the effect of the pH on anion removal, batch tests were conducted. The initial concentration of the phosphate, fluoride, and arsenic solution was selected as 12 mg L^−1^, 35 mg L^−1^, and 10 mg L^−1^, respectively, and the dosage was 0.2 g L^−1^. The pH of the solution was adjusted to 2.0–12.0 with 0.5 mol L^−1^ HCl solution and 0.5 mol L^−1^ NaOH solution. The adsorption capacity was calculated by the same method. Figure 6 shows that the pH of the solution had a great effect on the adsorption of anions, including the degree of ionization, the surface charge of the adsorbent, and the like. It can be seen that the adsorption capacity for fluoride removal increased with increasing pH and reached the maximum adsorption at pH 12, while the adsorption capacity for phosphate and arsenic removal reached its maximum at about pH 7. When the pH exceeds 7, the adsorption capacity decreases with increasing pH. This is because the inorganic pollutants themselves are affected by the pH value of the solution and form different ions. On the other hand, the surface charge of the adsorbent is also affected, resulting in reduced removal efficiency. Take the adsorption of PO_4_^3−^ in the pH 2–4 range as an example, where the driving force of adsorption is mainly electrostatic attraction. However, under alkaline conditions, the adsorption mechanism is via the ligand exchange between the hydroxyl group and phosphate [44]. When the solution’s pH is less than or equal to 2, Ce-MOF has no effect on anion adsorption because Ce-MOF has been completely dissolved, and there is zero point of charge at this time. 

#### 3.5.3. Effect of Reaction Time

**Figure 7:**
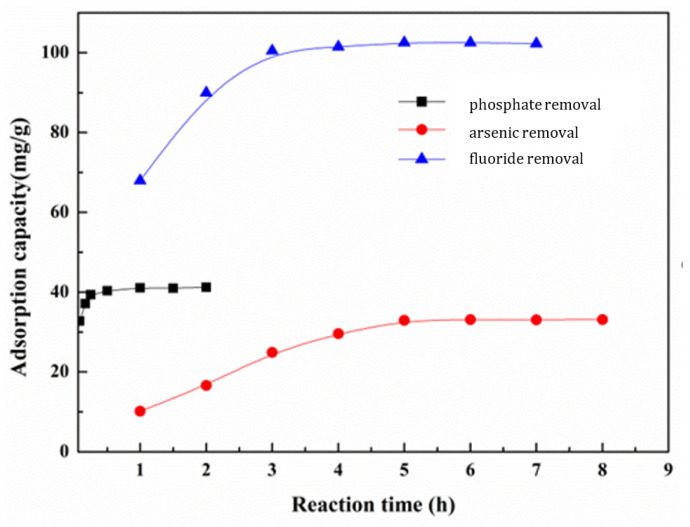
Effect of reaction time on phosphate, fluoride, and arsenic removal.

For the application of adsorbents in wastewater treatment, one of the important parameters is the determination of adsorption time. To explore the effect of the reaction time on phosphate, fluoride, and arsenic removal, we chose time as the reaction variable, ranging from 0.1 h to 8 h. The initial concentration of the phosphate, fluoride, and arsenic solution was selected as 12 mg L^−1^, 35 mg L^−1^, and 10 mg L^−1^, respectively, and the dosage was 0.2 g L^−1^, respectively. Figure 7 shows the adsorption capacity as a function of the reaction time. It can be seen that the adsorption amount of phosphate gradually increases with increasing adsorption time within 30 min due to the large affinity between the adsorbate and adsorbent surface in the phosphate solution. After that point, the adsorption process moves into a relatively gentle stage, where the adsorption capacity trend is very small and the adsorption process reaches equilibrium. The trend of the adsorption capacity for fluoride and arsenic removal with increasing reaction time is the same as phosphate removal. Therefore, when the reaction time of phosphate, fluoride, and arsenic removal were 30 min, 3 h, and 5 h, respectively, the reactions reached adsorption equilibrium.

#### 3.5.4. Adsorption Isotherm

The Langmuir adsorption isothermal model assumes uniformity on the surface of the adsorbent, where the adsorbed active sites are evenly distributed on the surface of the adsorbent and the energy of the adsorption centers on the adsorbent is the same and only a single adsorbate molecule is adsorbed [45]. It is apparent from the interaction that when the dynamic equilibrium is reached, the rate of adsorption and desorption is the same.

The formula is:

(3) $$q_{e}=\frac{q_{m}\;C_{e}}{K+C_{e}}$$

The formula for the conversion process can be calculated as:

(4) $$C_{e}=q_{m}\frac{C_{e}}{q_{e}}-K$$

where *q_e_* (mg g^−1^) is the equilibrium adsorption capacity, *q_m_* (mg g^−1^) is the saturated adsorption capacity, *C_e_* (mg L^−1^) is the equilibrium concentration, and *K* is the adsorption constant.

**Figure 8:**
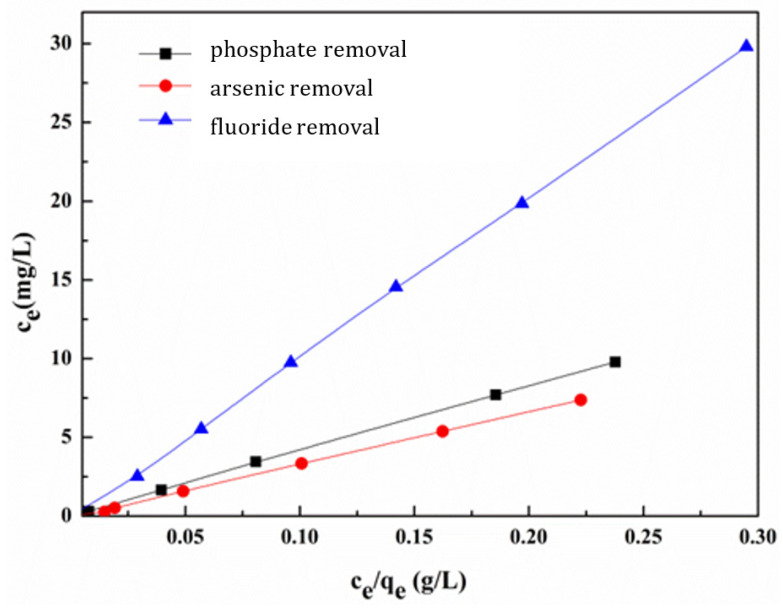
The Langmuir adsorption isothermal model of phosphate, fluoride, and arsenic removal.

Figure 8 shows the Langmuir isothermal adsorption curve of Ce-MOF for phosphate, fluoride, and arsenic removal, whose Langmuir fitting linear equations were *C_e_* = 41.24 *C_e_/q_e_* − 0.024 (R^2^ = 0.9999), *C_e_* = 33.29 *C_e_/q_e_* − 0.0083 (R^2^ = 0.9996), and *C_e_* = 101.76 *C_e_/q_e_* − 0.15 (R^2^ = 0.9998), which all have a high linear correlation coefficient. The theoretical maximum adsorption capacity of Ce-MOF for phosphate, fluoride, and arsenic removal were 41.2 mg g^−1^, 101.8 mg g^−1^ and 33.3 mg g^−1^, respectively, calculated by the Langmuir adsorption isotherm model. The results were consistent with the experimental equilibrium adsorption capacity. This indicates that the Langmuir isotherm is suitable for describing the adsorption behaviours of these anions’ removal. It also can be deduced that the adsorption of these anions over Ce-MOF is a type of adsorption of monomolecular layers.

The adsorption capacity of Ce-MOF for fluoride is much higher than that for phosphate and arsenic (V). This difference may be due to the fact that Ce can easily react with fluoride ions in solution through electrostatic adsorption and surface complexation, and fluoride is close to the hydroxyl radius (lower radius). In addition, the degree of complexation with the active site on the Ce surface is higher [46].

### 3.6. Comparison of Anion Removal Performance of Different Adsorbents

Under the same experimental conditions, the phosphate, fluoride, and arsenic (V) removal performances of Ce-MOF were compared with some commercially available adsorbents, including calcium salt, alum, hydrotalcite, and commercial CeO_2_ (Table 1), as well as other common adsorbents in the literature (Tables S4–S7). Again, there were very few reports about the removal of these three ions simultaneously. Obviously, the adsorption removal rates of Ce-MOF for phosphate, fluoride, and arsenic removal are all higher than those of other materials, reaching values of 98%, 96%, and 88%, respectively. In particular, compared with the commercial CeO_2_, Ce-MOF exhibited superior performance in the removal of these three ions. Even compared with the removal of single ions separately (Tables S5–S7), Ce-MOF demonstrates a higher removal rate than other MOFs. For instance, Ce-MOF shows higher adsorption properties than Fe_3_O_4_@NH_2_-MIL-101(Fe) for the removal of PO_4_^3−^ (Table S5). Interestingly, the adsorption capacity of Ce-MOF is ~2, 7.5, and 3 times higher than those of Ce-MIL-96, MOF-801, and UiO-66, respectively (Table S6). Ce-MOF also exhibits better removal rates than Fe-BTC, MOF-808, and MIL-53(Fe) (Table S7). Hence, Ce-MOF has an excellent ability to remove phosphate, fluoride, and arsenic from water. When facing future purification requirements, it can achieve a greater adsorption effect with less usage, reduce costs, and also meet the requirements of green production. Therefore, Ce-MOF is suitable for removing phosphate, fluoride, and arsenic (V) simultaneously from aqueous systems.

### 3.7. Adsorption Recyclability of Materials

Under the same experimental conditions, we performed two reuses and found that the performance of the adsorbent was almost unchanged, as shown in Table 2. SEM images showed that the adsorbent was nearly the same before and after the adsorption.

## 4. Conclusions

In this work, Ce-MOF was synthesized by a solvothermal synthesis method with a surface area of 222.4 m^2^ g^−1^. Our isotherm and kinetic studies suggest that the adsorption behaviour was consistent with the Langmuir isotherm model. The maximum adsorption capacity of phosphate, fluoride, and arsenic (V) removal was 41.2 mg g^−1^, 101.8 mg g^−1^, 33.3 mg g^−1^, respectively. The adsorption capacity of Ce-MOF is much higher than that of most commercial adsorbents. Particularly, Ce-MOF exhibits superior performance in the removal of these three ions compared with commercial CeO_2_. The pH of the solution had a great effect on the adsorption of anions. Furthermore, after two reuses, the performance of the adsorbent was almost unchanged, indicating it is a stable adsorbent and has good application potential in the field of wastewater treatment. 

## Figures and Tables

**Table 1:** The removal performance of different adsorbents (dosage 0.2 g L^−1^) for (1) phosphate: *C*_0_ = 15 mg L^−1^, t = 1 h; (2) fluoride: *C*_0_ = 15 mg L^−1^, t = 6 h; and (3) arsenic: *C*_0_ = 0.5 mg L^−1^, t = 6 h.

Adsorbents	Removal Rate (%)		
	Phosphate Removal (%)	Fluoride Removal (%)	Arsenic Removal (%)
Calcium salt	79	90	44
Alum	69	68	12
Hydrotalcite	76	72	24
Commercial CeO_2_	25	40	1
Ce-MOF	98	96	88

**Table 2:** Determination of adsorption rate after multiple adsorptions of the same adsorbent.

Adsorbents	Phosphate Removal (%)	Recovery (%)
First adsorption	98	-
Second adsorption	95	89
Third adsorption	91	74

## Data Availability

Data are contained within the article.

## Supplementary Materials

The following supporting information can be downloaded at: https://www.mdpi.com/article/10.3390/nano13233048/s1. Text S1; Figure S1. XRD patterns for CeO_2_; Table S1. Optimization and screening of solvent amount; Table S2. Screening of reaction temperature; Table S3. Optimization of reaction time and screening; Table S4. Comparison of adsorption capacity of some conventional adsorbents for fluoride, arsenate, and phosphate according to the literature; Table S5. Phosphate adsorption capacity of various materials [47]; Table S6. Fluoride adsorption capacity of various materials [48,49,50]; Table S7. Arsenic (V) adsorption capacity of various materials [51,52,53].
