# Supplementary material for: Facile Synthesis of Ce-MOF for the Removal of Phosphate, Fluoride, and Arsenic
Source: Nanomaterials (Basel). 2023 Nov 29;13(23):3048. doi: 10.3390/nano13233048 (PMC10707913; doi:10.3390/nano13233048)
Supplement: Supplementary file 1 [file nanomaterials-13-03048-s001.zip › nanomaterials-2721232-supplementary.pdf]

nanomaterials

Supplementary Materials:

Facile Synthesis of Ce-MOF for the Removal of Phosphate, Fluoride, and Arsenic

Lili Zhang [1,†], Decheng Mao [2,†], Yining Qu [2], Xiaohong Chen [2], Jindi Zhang [1], Mengyang Huang [1,*] and Jiaqiang Wang [1,2,*]

Text S1

All reagents were of analytical grade. The reagents used included sodium phosphate ($NaH_2PO_4·2H_2O$, AR, Tianjin Fengchuan Chemical Technologies Co., Ltd), sodium fluoride (NaF, AR, Chongqing Chuandong Chemical Group Co., Ltd), arsenic(V) (H5As3O10, AR, Chongqing Chuandong Chemical Group Co., Ltd), 1,3,5-Benzenetricarboxylic acid (H3BTC, Sinopharm Chemical Reagent Co., Ltd, ≥99%), terephthalic acid (H2BDC, Sinopharm Chemical Reagent Co., Ltd, ≥99%), cerium nitrate ($Ce(NO_3)_3·6H_2O$, Tianjin Guangfu Fine Chemical Research Institute, ≥99%), cerium chloride ($CeCl_3·6H_2O$, Tianjin Guangfu Fine Chemical Research Institute, ≥99%), cerium sulfate ($Ce_2(SO_4)_3·8H_2O$, Tianjin Guangfu Fine Chemical Research Institute, ≥99%), N,N-dimethyl-formamide (DMF, AR, Xilong Chemical Co., Ltd), and ethanol (AR, Xilong Chemical Co., Ltd).

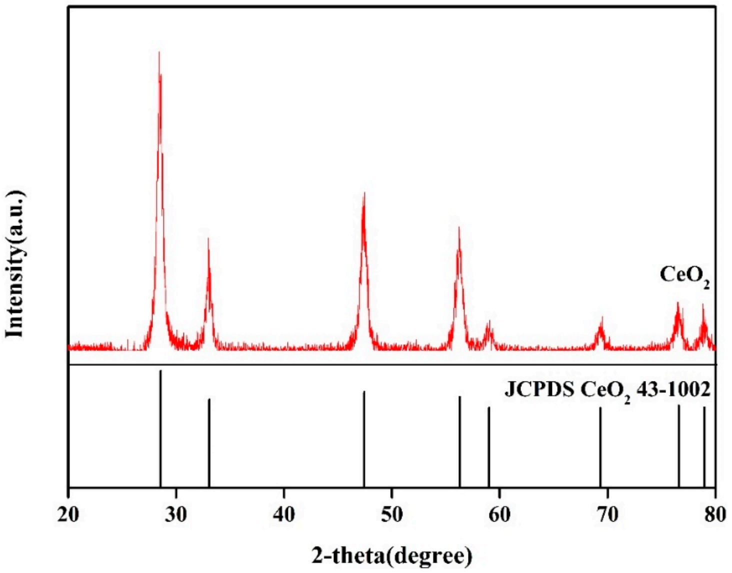

Figure S1. XRD patterns for CeO_2.

Table S1. Optimization and screening of solvent amount.

DMF（mL）	Phosphate removal rate after 1 h
100	77.7%
80	89.8%

60	92.5%
40	78.9%

Table S2. Screening of reaction temperature.

Temperature ℃	Phosphate removal rate after 1 h
200	87.9%
180	97.5%
150	91.7%
100	67.2%

Table S3. Optimization of reaction time and screening.

Time	Phosphate removal rate after 1 h
4d	96.8%
3d	97.9%
2d	89.8%
1d	77.2%

Table S4. Comparison of adsorption capacity of some conventional adsorbents for fluoride, arsenate, and phosphate according to the literature.

Adsorbents	Adsorption capacity (mg g^{-1})			Reference
	Phosphate	Fluoride	Arsenic	
Mg–Al–Fe layered double hydroxide/polyether sulfone-mixed matrix membranes	5.61	1.61	—	[1]
Modified saxaul ash	—	—	4.203	[2]
Magnetic particles sprayed with gelatin with embedded hydrotalcite composite Fe_3O_4 @GelHT	32.73	—	—	[3]
Calcined La-doped layered double hydroxides	—	62.33	87.3	[4]

Ce-MOF	41.2	101.8	33.3	This study

Table S5. Phosphate adsorption capacity of various materials.

Adsorbent	pH	Adsorption capacity (mg g^{-1})	Ref.
Fe_3O_4@NH_2-MIL-101(Fe)	7.0	36.6	[5]
Ce-MOF	7.0	41.2	This study

Table S6. Fluoride adsorption capacity of various materials.

Adsorbent	pH	Adsorption capacity (mg g^{-1})	Ref.
Ce-MIL-96	3-10	52.1	[6]
MOF-801	2.0	13.6	[7]
UiO-66	6.8	33.4	[8]
Ce-MOF	7.0	101.8	This study

Table S7. Arsenic(V) adsorption capacity of various materials.

Adsorbent	pH	Adsorption capacity (mg g^{-1})	Ref.
Fe-BTC	2-10	12.3	[9]
MOF-808	4.0	13.6	[10]
MIL-53(Fe)	5.0	21.3	[11]
Ce-MOF	7.0	33.3	This study

References

1. Pintor, A.M.; Vilar, V.J.; Botelho, C.M.; Boaventura, R.A. Oil and grease removal from wastewaters: Sorption treatment as an alternative to state-of-the-art technologies. A critical review. *Chem. Eng. J.* **2016**, *297*, 229–255.
2. Liu, X.; Yu, X.; Sha, L.; Wang, Y.; Zhou, Z.; Zhang, S. The preparation of black titanium oxide nanoarray via coking fluorinated wastewater and application on coking wastewater treatment. *Chemosphere* **2021**, *270*, 128609.
3. Rosales, M.; Coreño, O.; Nava, J.L. Removal of hydrated silica, fluoride and arsenic from groundwater by electrocoagulation using a continuous reactor with a twelve-cell stack. *Chemosphere* **2018**, *211*, 149–155.
4. Rahdar, S.; Taghavi, M.; Khaksefidi, R.; Ahmadi, S. Adsorption of arsenic (V) from aqueous solution using modified saxaul ash: Isotherm and thermodynamic study. *Appl. Water. Sci.* **2019**, *9*, 87.
5. Zhou, R.Y.; Yu, J.X.; Chi, R.A. Selective removal of phosphate from aqueous solution by MIL-101 (Fe)/bagasse composite prepared through bagasse size control. *Environ. Res.* **2020**, *188*, 109817.
6. Yang, X.; Deng, S.; Peng, F.; Luo, T. A new adsorbent of a Ce ion-implanted metal–organic framework (MIL-96) with high-efficiency Ce utilization for removing fluoride from water. *Dalton Trans.* **2017**, *46*, 1996–2006.
7. Zhu, X.; Yang, C.; Yan, X. Metal-organic framework-801 for efficient removal of fluoride from water. *Microporous Mesoporous Mater.* **2018**, *259*, 163–170.
8. Zhao, X.; Liu, D.; Huang, H.; Zhang, W.; Yang, Q.; Zhong, C. The stability and defluoridation performance of MOFs in fluoride solutions. *Microporous Mesoporous Mater.* **2014**, *185*, 72–78.

9. Zhu, B.; Yu, X.; Jia, Y.; Peng, F.; Sun, B.; Zhang, M.; Huang, X. Iron and 1, 3, 5-benzenetricarboxylic metal–organic coordination polymers prepared by solvothermal method and their application in efficient As (V) removal from aqueous solutions. *J. Phys. Chem. C* **2012**, *116*, 8601–8607.
10. Li, Z.; Yang, J.; Sui, K.; Yin, N. Facile synthesis of metal-organic framework MOF-808 for arsenic removal. *Mater. Lett.* **2015**, *160*, 412–414.
11. Vu, T.A.; Le, G.H.; Dao, C.D.; Dang, L.Q.; Nguyen, K.T.; Nguyen, Q.K.; Lee, G.D. Arsenic removal from aqueous solutions by adsorption using novel MIL-53 (Fe) as a highly efficient adsorbent. *RSC Adv.* **2015**, *5*, 5261–5268.
